# Risk factors for visual field progression during 10-year follow-up in newly diagnosed exfoliation glaucoma patients

**DOI:** 10.1038/s41598-026-60254-x

**Published:** 2026-06-30

**Authors:** Marcelo Ayala, Tobias Dahlgren

**Affiliations:** 1 Eye Department, Mölndal Hospital, Sahlgrenska University Hospital, Region Västra Götaland, Göteborgsvägen 31, Gothenburg, Mölndal, 431 80 Sweden; 2 Department of Ophthalmology, NU Hospital Group, Region Västra Götaland, Uddevalla, Sweden; 3 Department of Clinical Neuroscience, Institute of Neuroscience and Physiology, Sahlgrenska Academy, University of Gothenburg, Gothenburg, Sweden

**Keywords:** Cohort studies, Exfoliation glaucoma, Intraocular pressure, Models, Visual fields, Diseases, Medical research, Risk factors

## Abstract

To identify risk factors associated with visual field progression over long-term follow-up (10 years) in patients newly diagnosed with exfoliation glaucoma. This was a non-randomised, prospective cohort study including patients newly diagnosed with exfoliation glaucoma. Visual field progression was assessed using three methods mean deviation (MD), visual field index (VFI), and guided progression analysis (GPA). Baseline variables to be evaluated included intraocular pressure (IOP), sex, age, visual fields, uni- or bilateral presentation, visual acuity, lens status (phakic/pseudophakic), central corneal thickness (CCT), optic nerve status, and gonioscopy findings. Additionally, a questionnaire on, hypertension, diabetes, migraine, smoking, and family history of glaucoma. Multivariable regression analyses were performed to identify risk factors. A total of 58 patients completed the 10-year follow-up. The IOP at inclusion was 32.06 (± 6.05) mmHg. In the MD model, significant risk factors for progression included older age (*p* = 0.002), smoking (*p* = 0.004), phakic lens status (*p* < 0.001), and lower CCT (*p* = 0.02). In the VFI model, significant risk factors were older age (*p* = 0.009) and smoking (*p* = 0.04). In the GPA model, risk factors included older age (*p* = 0.001) and smoking (*p* = 0.01). Older age at diagnosis and a history of smoking were found to be significantly associated with an increased risk of long-term visual field progression.

**Supplementary Information:**

The online version contains supplementary material available at 10.1038/s41598-026-60254-x.

## Introduction

Glaucoma is a common eye disease. The disease is characterised as a chronic, progressive, potentially blinding, irreversible condition that causes loss of the optic nerve rim and retinal nerve fibre layer, resulting in visual field defects^1^. In Sweden, primary open-angle glaucoma (POAG) and exfoliative glaucoma (EXFG) are the most common types of glaucoma^2^. The EXFG is caused by the accumulation of protein fibrillar material in the eye’s trabecular meshwork. This build-up diminishes the outflow of aqueous humour, thereby increasing intraocular pressure (IOP). The reason why EXFG is so common in Scandinavia remains unknown, but several genes have been identified as related to the condition^3–5^. Several studies suggest that EXFG is a rapidly progressive type of glaucoma. Visual field deterioration is reported to be at least three times faster in EXFG than in POAG^6–8^.

According to the Swedish guidelines for glaucoma care, at least six visual fields are required during the two years after diagnosis to evaluate the progression rate. Then, the number of visual fields can be reduced to 1–2 per year^9^. Visual fields remain the most effective way to detect progression in glaucoma. Since visual field performance can vary within a subject, multiple tests are required to establish disease progression. Anatomical measurements of the optic nerve using the Optical Coherence Tomograph (OCT) are employed in clinical practice. However, OCT has its drawbacks and can be considered a complement to visual fields in assessing glaucoma progression^1^. Evidence regarding long-term visual field progression in glaucoma studies is limited because patients are usually elderly at inclusion and cannot be observed over many years. Moreover, studies that focus solely on newly diagnosed glaucoma patients are rare, as most general glaucoma studies include both newly and previously diagnosed patients. Furthermore, exfoliation glaucoma patients are uncommon in other populations and have been excluded from several large cohort studies^10–12^.

The present study aimed to identify risk factors for long-term (10-year) visual field progression in patients newly diagnosed with exfoliation glaucoma.

## Materials and methods

This was a non-randomised prospective observational cohort study that included patients newly diagnosed with EXFG. The study recruited patients who attended the Ophthalmology Department at Skaraborg Hospital, Skövde, Sweden, from 1 January 2013 to 31 December 2015. All patients received oral and written information before being included in the study and signed the informed consent form. The Regional Ethical Review Board in Gothenburg granted ethical approval for the study (DN:119 − 12). The study adhered to the principles of the Helsinki Declaration. The present study was conducted on a cohort gathered for the purpose of genetic analysis and subsequently followed for ten years to assess clinical progression. All research activities were carried out in collaboration with the University of Gothenburg.

### Inclusion criteria

1. Patients newly diagnosed with EXFG, according to the European Glaucoma Society (EGS) guidelines.
2. Age ≤ 85 years.

### Exclusion criteria

1. Patients unable to perform reliable visual field tests (e.g., due to dementia, poor cooperation, or advanced glaucoma). Reliable visual fields were defined as those displaying false positives ≤ 10% and false negatives ≤ 20%.
2. Patients showing advanced visual field damage at diagnosis, defined as mean deviation (MD) ≤-20 dB.
3. Patients with other eye conditions alongside glaucoma that could affect visual fields, such as central venous occlusion, retinal degeneration, etc.
4. Patients who died before 10 years had passed, or could not be followed due to dementia, general illness, or because they relocated to another part of the country.
5. Patients who underwent glaucoma surgery, such as trabeculectomy, shunt surgery, or transscleral cyclophotocoagulation were excluded. The reason for exclusion was to eliminate confounding factors that make it difficult to measure the natural progression of the disease. Surgery alters the eye’s physiology, leading to differences in data compared with patients treated with medication. Patients who had uncomplicated cataract surgery or selective laser trabeculoplasty (SLT) were not excluded from the study.

All patients were referred to the Ophthalmology Department by optometrists due to high IOP (≥ 25 mmHg) and attended a recruitment visit. During this visit, patients completed a questionnaire and underwent basic ophthalmological examinations. An assistant ophthalmic nurse first assisted the patients with filling in the questionnaire. The questionnaire collected information on blood pressure, diabetes, migraine, smoking, medications, family history of glaucoma, and other systemic factors. After completing the questionnaire, the patients’ age and sex were recorded. Subsequently, the best-corrected visual acuity was measured by an assistant ophthalmic nurse using a Snellen chart. Subsequently, the assistant nurse performed visual field tests. The Humphrey Field Analyser (Carl-Zeiss, Straße 22, 73447 Oberkochen, Germany) was employed to evaluate visual fields using the fast 24 − 2 Swedish Interactive Threshold Algorithm (SITA) fast.

The patients were then examined by an ophthalmologist (MA). The IOP was measured three times using a Goldmann applanation tonometer; the average value was calculated and recorded. The central corneal thickness (CCT) was measured with an ultrasound device (Tomey Pachymetry; Tomey Corp., Nagoya 451–0051, Japan). Gonioscopy was performed with a goniolens on undilated pupils to inspect the trabecular meshwork.

The patient’s pupils were dilated. Exfoliation was observed in the eye’s anterior part with a slit lamp. A 90-D lens was used to examine the optic nerve, and the cup-to-disc (C/D) ratio was noted. If unilateral EXFG was present, that eye was included. If both eyes had EXFG, one was randomly selected for the study.

All patients received treatment with IOP-lowering eye drops at inclusion. The IOP was measured one month after the recruitment appointment to evaluate the medication’s effect. Patients were followed for ten years. In the first three years (± 1 month), patients underwent examinations every six months (± 1 month). Visual acuity, IOP, and visual fields were assessed at each visit. After three years, all patients were followed at least once per year for ten years. If patients performed more than one visual field test per year, the average result was calculated for analysis. Decisions on patient management were based on the Swedish Guidelines for glaucoma care^9^.

During the ten years of follow-up, several patients’ IOP required additional treatment. Treatment escalation involved increasing the number of medicines or performing selective laser trabeculoplasty (SLT). For analysis purposes, the number of IOP-lowering eyedrops was classified by the number of substances, not the number of bottles used. For SLT treatment, it was recorded as Yes/No. The same approach was applied to cataract surgery.

### Endpoints

The study’s primary objective was to investigate risk factors for visual field progression in newly diagnosed EXFG patients over a long-term period (ten years). Visual field progression was measured in three different ways. Two methods assessed progression continuously, while the third used a binary approach.

The first continuous method was based on the mean deviation (MD) values of visual fields. Although MD is the oldest method for measuring visual field progression, it is still in use, and the well-known classification by Hodapp et al.^13^ was based on MD values. In the present study, the difference between the MD values at the beginning (inclusion) and at the end of the study was calculated and used as the endpoint for visual field progression.

The second continuous method employed was the visual field index (VFI), which is widely used in clinical practice. The device calculates the VFI as a percentage, with a normal visual field corresponding to 100%. In the present study, the difference in the values from the inclusion to the end of the study was calculated and used as the endpoint for progression.

Finally, the binary method used was the glaucoma progression analysis (GPA). The GPA compares the value in each point with the corresponding points in earlier assessments and categorises progression as none, possible, or likely. In this study, GPA outcomes were classified as progressing or not progressing (binary variables) at the final (10 years) measurement, with progression encompassing both “possible” and “likely” cases.

### Statistics

The IBM SPSS software (Armonk, NY 10504, USA) was used to conduct statistical analyses. All tests were two-tailed, *p* < 0.05 was the limit for statistical significance (unless otherwise stated), and 95% confidence intervals were used. Descriptive statistics were expressed as “mean (± standard deviation)” for continuous variables, “median (interquartile range)” for ordinal variables and “number of cases (percentage)” for discrete variables. The Kolmogorov-Smirnov test was used to assess the normality of MD and VFI values. Regression analysis was performed to evaluate the impact of several variables on progression. For continuous dependent (outcome) variables (MD and VFI), linear regression was utilised. For the binary dependent variable (GPA), logistic regression was applied.

All variables (predictors) were analysed using a “two-step model”. The first step involved testing all potential predictors using a single-variable regression model. Variables with *p* ≤ 0.10 were then included in a multivariable regression model^14^. The significance level for multivariable regressions was set at *p* < 0.05.

## Results

Altogether, 58 patients were followed for ten years. Initially, 92 patients were recruited at the first visit (inclusion visit). Patients (*n* = 34) were excluded for various reasons. The main reason for exclusion was that patients died (*n* = 12) or were in such poor condition (*n* = 5) that they could not attend follow-up visits. Among those who died, 3 deaths were due to Covid-19 infection.

The second reason for exclusion was glaucoma surgery; 5 patients had trabeculectomy, and 2 had shunt surgeries. The average age for surgery was 76.83 ± 2.63 years. Surgery was performed 4.91 ± 0.8 years after the diagnosis. Among patients who were operated on, four patients were smokers, and three were not. The reason for surgery in all cases was uncontrolled IOP.

**Fig. 1:**
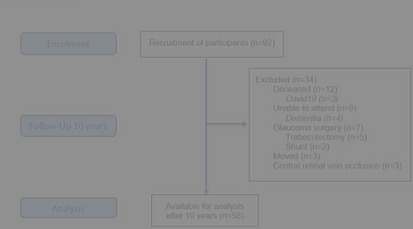
Flow-chart. The figure shows the flow-chart during the 10-year follow-up

The third reason was dementia, as 4 patients developed dementia and could not participate in visual field testing. The fourth reason involved patients who moved (*n* = 3) and the fifth reason patients who developed a central retinal vein occlusion (CRVO) (*n* = 3). Please see Fig. 1 for more details.

The patients included in this study had an average age at inclusion of 71.24 ± 7.22 years. There was an almost equal distribution between sexes: 27 males and 31 females (47/53%). The median visual acuity in Snellen units was 0.9 (0.68-1), and the mean intraocular pressure (IOP) at inclusion was 32.06 (± 6.05) mmHg. The glaucoma presentation was unilateral in 39 cases and bilateral in 19 cases (67/33%). The mean central corneal thickness (CCT) was 542.6 (± 39.19) µm. The average cup/disc ratio was 0.74 (± 0.16). Only 18 out of 58 patients (31%) had undergone cataract surgery (pseudophakia) at the time of inclusion. Regarding smoking, 21 patients were categorized as smokers when included, but only 2 of these were smoking at the time of inclusion, the rest (*n* = 19) were “former smokers”. For more details regarding baseline characteristics, please see Table 1.

**Table 1:** Clinical characteristics of the cohort at baseline.

Clinical characteristics	*N* = 58
Age (years) (SD)	71.24 ± 7.22
Sex (M/W) (%)	27/31 (47/53)
IOP at diagnosis (mmHg) (SD)	32.06 ± 6.05
Visual acuity (Snellen)*	0.9 (0.68-1)
Unilateral/Bilateral (%)	39/19 (67/33%)
Gonioscopy (Shaeffer) (SD)	3.20 ± 0.52
Gonioscopy (pigmentation) (SD)	2.55 ± 0.53
CCT (µm) (SD)	542.60 ± 39.13
C/D ratio (SD)	0.74 ± 0.16
RNFL OCT (µm) (SD)	66.27 ± 20.58
Phakic/pseudophakic (%)	40/18 (69/31)
Family history (Yes/No) (%)	25/33 (43/57)
Diabetes (Yes/No) (%)	10/48 (17/83)
Hypertension (Yes/No) (%)	29/29 (50/50)
Smoking (Yes/No) (%)	21/37 (36/64)
Migraine (Yes/No) (%)	5/53 (9/91)
MD at diagnosis (dB) (SD)	− 5.38 ± 4.37
VFI at diagnosis (%) (SD)	87.46 ± 12.12

*IOP* intraocular pressure, *CCT* central corneal thickness, *C/D ratio* cup/disc ratio, *RNFL* retinal nerve fiber layer, *OCT* optical coherence tomograph, *MD* mean deviation, *VFI* visual field index

*Median values and 25–75 quartiles in brackets

Patients were divided into progressors and non-progressors at baseline based on GPA. The baseline characteristics are shown in Table 1 (supp.).

All patients were monitored for 10 years during the study, with a ± 3-month margin.

**Fig. 2:**
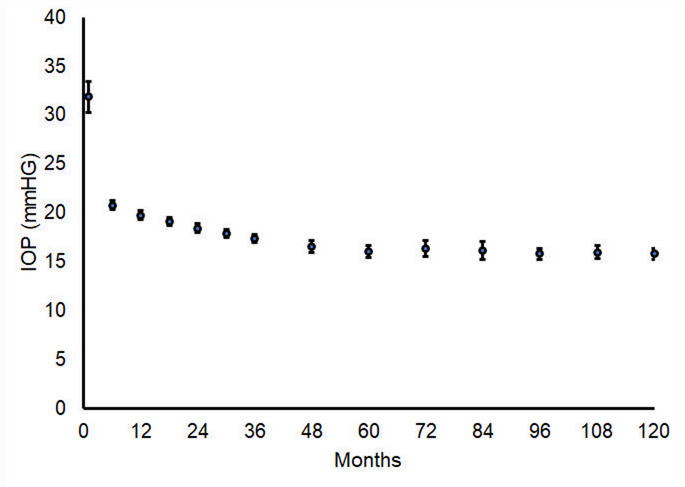
Evolution of the IOP values in the ten-year follow-up period. The bars represent the 95% confidence interval for the mean.

The mean IOP at the time of inclusion was 32.06 ± 6.05 mmHg. At six months, the average IOP value was 20.79 ± 1.88 mmHg. By the end of the study (ten years), the mean IOP was 15.71 ± 2.07 mmHg. For more details, please see Fig. 2.

**Fig. 3:**
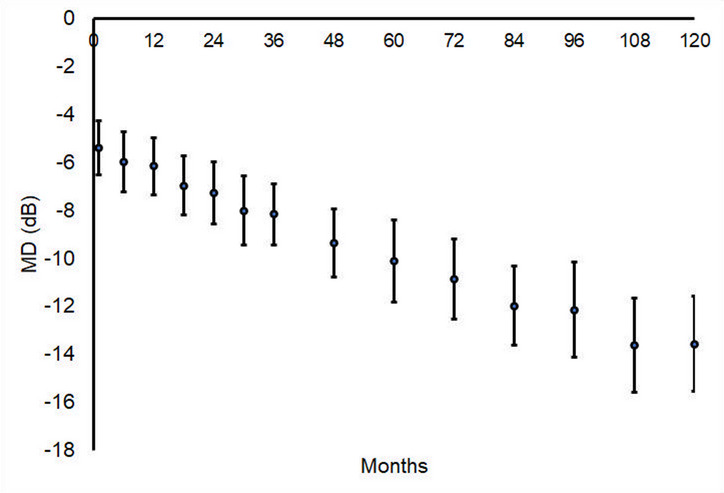
Evolution of the MD values in the ten-year follow-up period. The bars represent the 95% confidence interval for the mean.

The average MD value at the start of the study was − 5.38 ± 4.37 dB. The mean decline in MD was − 7.94 ± 6.37 dB over the 10-year study period. The annual slope of MD values and the proportion of non-progressors and progressors based on MD values are shown in Table 2, Supplement. By the end of the study (ten years), the average value was − 13.54 ± 7.84 dB. For more details, see Fig. 3.

**Fig. 4:**
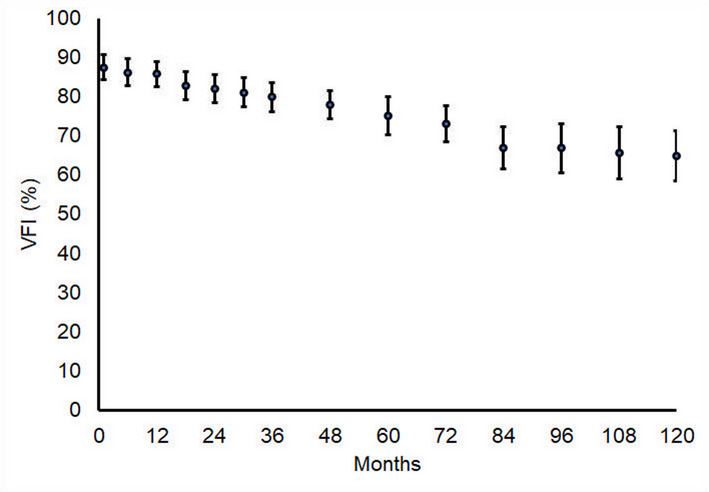
Evolution of the VFI values in the ten-year follow-up period. The bars represent the 95% confidence interval for the mean.

The average VFI value at the start of the study was 87.46 ± 12.12%. The mean deterioration of the VFI was 22.63 ± 22.51% over 10 years. By the end of the study, the average VFI value was 65 ± 24.37%. For more details, see Fig. 4.

During the study period, the patients underwent GPA evaluation, which categorised the group into two: those who showed progression and those who did not. In total, 20 (34%) patients did not experience visual field deterioration, while 38 (66%) did.

Over ten years, cataract surgery was performed on 32 patients. At baseline, 40 patients were phakic, indicating that during the follow-up period, the majority underwent cataract surgery: 32 of 40 (80%). By the end of the study, 50 of 58 patients (86%) were pseudophakic. The average number of medications at the end of the study was 2.89 ± 1.28. During the follow-up period, 25 patients (43%) received at least one SLT treatment.

To assess risk factors, all predictors were initially tested in separate regression models. The variables with *p* < 0.10 were then included in a multivariable model. For the single-variable models, please see Table 3 supp, Table 4 supp, and Table 5 supp.

The risk factors for visual field progression in the MD multiple linear regression model were age (*p* = 0.002), CCT (*p* = 0.02), phakic status (p = < 0.001), and current or previous smoking (*p* = 0.004). The IOP value at diagnosis was not a significant risk factor (*p* = 0.49). The higher the age, the greater the progression in MD values. CCT values were negatively associated with progression; lower values were associated with progression. Concerning phakia/pseudophakia at inclusion, patients who were phakic at the start showed increased progression over the ten-year follow-up period. However, cataract surgery during the 10-year follow-up period did not alter visual field progression. Smokers exhibited more visual field progression than non-smokers. For more details, please see Table 2.

**Table 2:** Multiple linear regression analysis for predictors, with the MD value after ten years of follow-up as the dependent variable.

Predictor	Uns. Coeff. B	Coefficient 95% CI	Stand. Coeff.B	*P*-value
Age	0.30	[0.12;0.49]	0.35	0.002*
CCT	− 0.04	[− 0.07;-0.007]	− 0.26	0.02*
IOP at diagnosis	0.08	[− 0.15;0.30]	0.08	0.49
Pseudophakia	− 5.7	[− 8.58; − 2.82]	− 0.42	< 0.001*
Smoking	4.22	[1.44;6.99]	0.32	0.004*

*CCT* central corneal thickness, *IOP* intraocular pressure

*Significant values *p* ≤ 0.05

Regarding risk factors for progression based on VFI measurements, the multiple linear regression analysis indicated that age (*p* = 0.009) and smoking (*p* = 0.004) were significantly linked to progression. However, IOP at diagnosis and CCT were not significant in this model. As mentioned above, higher age was associated with greater VFI progression. Smokers showed a higher degree of VFI progression than non-smokers. For more details, please see Table 3.

**Table 3:** Multivariate analysis for predictors, endpoint VFI values across ten years-follow-up.

Predictor	Uns. Coeff. (B)	Coefficient 95 CI	Stand. Coeff. B	*P*-value
Age	0.94	[0.25;1.64]	0.31	0.009*
CCT	− 0.12	[− 0.25;0.008]	− 0.22	0.07
IOP at diagnosis	0.73	[− 0.11;1.58]	0.20	0.09
Smoking	11	[0.57;11.49]	0.24	0.04*

*CCT* central corneal thickness, *IOP* intraocular pressure

*Significant values *p* ≤ 0.05

The third model assessed visual field progression as a binary (yes/no) variable using the GPA analysis. Age (*p* = 0.001) and smoking (*p* = 0.01) were significant predictors of progression. However, IOP at diagnosis and CCT were not significant predictors for progression based on GPA. For further details, please see Table 4.

**Table 4:** Logistic multivariate analysis for predictors, endpoint GPA values across ten years-follow-up.

Predictor	Odds Ratio (OR)	OR 95% CI	*P*-value
Age	1.3	[1.11;1.53]	0.001*
CCT	1	[0.98;1.02]	0.86
IOP at diagnosis	1.14	[0.99;1.32]	0.06
Smoking	23	[1.87;284.81]	0.01*

*CCT* central corneal thickness, *IOP* intraocular pressure

*Significant values *p* ≤ 0.05

The logistic multivariable analysis showed a strong relationship between the predictor variables and the outcomes. (Nagelkerke r square = 0.63).

## Subgroup analysis

The whole cohort was divided into two groups based on MD values of < 1 dB/year and ≥ 1 dB/year: no progressors and progressors. A logistic regression analysis was performed to identify risk factors. The risk factors for progression were age (*p* = 0.03) and smoking (*p* = 0.04). Please see Table 5.

**Table 5:** Logistic multivariate analysis for predictors, endpoint according to MD values as progressors and no progressor across ten years-follow-up.

Predictor	Odds Ratio (OR)	OR 95% CI	*P*-value
Age	1.12	[1;1.26]	0.03*
CCT	0.98	[0.96;1]	0.15
IOP at diagnosis	1.06	[0.95;1.2]	0.28
Smoking	3.2	[0.78;13.20]	0.04*
Pseudophakia	0.3	[0.06;1.49]	0.14

*CCT* central corneal thickness, *IOP* intraocular pressure

*Significant values *p* ≤ 0.05

## Discussion

The present analyses demonstrated that increased age and smoking are key risk factors for long-term visual field progression in newly diagnosed exfoliation glaucoma patients. These two factors proved to be significant across all three models studied. Additionally, lower CCT and phakic status were significant in the MD model. Interestingly, the IOP at diagnosis was not a long-term risk factor. IOP at diagnosis has been described as a risk factor in three-year models^15,16^, but it was not significant in long-term models. In this study, the damaging effect of high baseline IOP (at diagnosis) declined over time, which is indeed the purpose of individualised glaucoma treatment. Progression rates would have been higher, and several patients might have gone blind, had they not received treatment during the ten years following the EXFG diagnosis.

Age has been identified as a risk factor in previous studies^16^. We should closely monitor older patients, as they are more likely to develop more severe visual field defects than younger patients. Conversely, younger patients are expected to live longer and must be protected from significant glaucoma progression for a longer time. In the present study, a significant number of patients died or were in very poor condition (*n* = 17/18%) during follow-up, which introduced some bias into the results.

Smoking was a risk factor in all three models studied. In the present study, the vast majority of the patients were “former smokers”, with only two patients smoking at the time of inclusion. Most smoked in the age range of 20–40 and consumed around 10 cigarettes per day. No “heavy smokers” were among the included patients. Probably, the association between smoking and visual field progression in these patients was due to alterations in blood flow in the optic nerve or the retina. Interestingly, prior or current smoking was a significant predictor, even though only two patients were still smoking when the study started. Smoking probably caused permanent vascular changes that made the eyes more vulnerable even after the patients had stopped smoking. Other variables related to smoking, such as social factors, education, and income, were not included in this study. However, Sweden is considered a relatively egalitarian society with universal access to healthcare; thus, it is unlikely that social factors had a significant impact on the results. Therefore, it is probable that smoking itself was the main cause of the observed visual field progression in this cohort.

Risk factors in the MD model also included lower CCT and phakic status. While the association between lower CCT and increased risk of progression is well established in glaucoma in general^17^, the literature provides limited evidence specifically addressing newly diagnosed exfoliation glaucoma. Therefore, our study contributes valuable new insights regarding these risk factors in this patient population. Phakic status was identified as a risk factor for progression. Phakic patients at inclusion showed an increased visual field progression than patients who were pseudophakic at inclusion. However, this finding should be interpreted with caution, as patients who had undergone cataract surgery exhibited lower IOP compared to those who remained phakic. Cataract surgery is a well-recognised intervention for reducing IOP in patients with exfoliation glaucoma^18^. Additionally, it is possible that cataract surgery facilitates the mechanical removal of exfoliation material, thereby reducing IOP and the risk of progression prior to study inclusion.

The GPA analysis identified smoking as a strong risk factor for progression, with logistic regression yielding an odds ratio (OR) of 23. However, these findings should be interpreted with caution, as logistic regression with two binary variables—both the predictor and the endpoint—can yield inflated OR estimates. The GPA method approximates an underlying biological process; since glaucoma is a chronic, progressive disease, its progression is continuous rather than binary. Despite these methodological considerations, it remains prudent to advise glaucoma patients to stop smoking in order to reduce the risk of disease progression.

Numerous studies have investigated various risk factors associated with the development and progression of glaucoma^7,19,20^. Nevertheless, creating robust predictive models for glaucoma progression remains challenging, primarily due to substantial inter-individual variability and heterogeneous progression patterns across glaucoma subtypes. Additional complexities arise from inconsistencies in data-collection methodologies and differences in follow-up durations across studies. The generalizability of predictive models is further limited by difficulties in achieving external validity, particularly given the diverse characteristics of patient populations in glaucoma research. Despite these challenges, the present study provides important new insights into glaucoma progression, which may inform and facilitate the future development of more accurate predictive models.

This study has several limitations. Glaucoma progression was assessed exclusively using visual field testing, which, although considered the gold standard, does not provide anatomical measurements of the optic nerve, such as those obtained by Optical Coherence Tomography (OCT). Patients who underwent cataract surgery and/or SLT could have IOP spikes in the short term. This information was not collected in the patients’ records. All participants had exfoliation glaucoma, restricting the generalizability of the findings to other glaucoma subtypes. Additionally, as all patients were born in Sweden, potential genetic factors may have influenced the results, further limiting the applicability to populations with different genetic backgrounds. Patients with advanced glaucoma damage were excluded due to difficulties in assessing glaucoma progression in this group; thus, the findings are pertinent only to early or moderate glaucoma and should not be extrapolated to advanced disease. Selection bias is also a consideration, as patients who were unable to reliably perform visual field tests, or who could not complete follow-up due to other diseases or general health issues, were excluded. In all three analyses, we found a correlation between age and glaucoma progression. However, it is possible that slightly higher IOP levels or a higher progression rate might have been accepted (i.e., not prompted escalation) in older patients due to their shorter life expectancy. While appropriate from a holistic clinical perspective, this may have introduced bias to the results.

Furthermore, since patients who underwent glaucoma surgery were not included, the true proportion of progressing patients may be underestimated, despite the observation that two-thirds of patients exhibited rapid progression. Further, the long follow-up duration (10 years) raises the possibility that ageing and cognitive decline may have affected visual field performance. Finally, the present analyses were performed post hoc, and we advise that significant findings are evaluated in independent studies.

In conclusion, the current study indicates that age and smoking are significant risk factors for long-term visual field decline in newly diagnosed exfoliation glaucoma patients. Older patients should be monitored more frequently to detect progression, and it may be adequate to lower the IOP further, despite old age. Patients should be advised to stop smoking to avoid further increased risk of progression, even though the risk may remain at a higher level compared to non-smokers. Other suggested risk factors include phakic status and lower CCT. These two factors should be considered in the risk assessment at the time of diagnosis.

## Supplementary Information

Below is the link to the electronic supplementary material.

Supplementary Material 1

Supplementary Material 2

Supplementary Material 3

Supplementary Material 4

Supplementary Material 5

## Data Availability

The datasets generated during and/or analysed during the current study are available from the corresponding author on reasonable request.
