# Supplementary material for: Risk factors for visual field progression during 10-year follow-up in newly diagnosed exfoliation glaucoma patients
Source: Sci Rep. 2026 Jun 30;16:19909. doi: 10.1038/s41598-026-60254-x (PMC13319220; doi:10.1038/s41598-026-60254-x)
Supplement: Supplementary file 1 — Supplementary Material 1 [file 41598_2026_60254_MOESM1_ESM.docx]

Table 1-Supp.

Baseline characteristics of the predictors in the GPA model.

| ***Predictor*** | ***Non-progressors***  ***N=19*** | ***Progressors***  ***N=39*** | ***Test*** | ***P*** |
| --- | --- | --- | --- | --- |
| ***Age (years)*** | 69.94 ± 5.94 | 71.76 ± 6.12 | T-test | 0.04* |
| ***Sex (M/W) (%)*** | 7/12 (37/63%) | 16/23 (41/59%) | Chi-sq | 0.57 |
| ***IOP at diagnosis (mmHg)*** | 28.42 ± 4.58 | 33.48 ± 5.34 | T-test | <0.001* |
| ***Visual acuity*** | 0.84 ± 0.22 | 0.81 ± 0.21 | T-test | 0.60 |
| ***Unilateral/Bilateral (%)*** | 13/6 (68/32%) | 26/13 (66/34%) | Chi-test | 0.92 |
| ***Gonioscopy (Shaeffer)*** | 3.05 ± 0.52 | 3.28 ± 0.51 | T-test | 0.12 |
| ***Gonioscopy pigmentation*** | 2.57 ± 0.61 | 2.53 ± 0.55 | T-test | 0.80 |
| ***CCT (µm)*** | 549 ± 48 | 539 ± 34 | T-test | 0.38 |
| ***C/D ratio*** | 0.75 ± 0.11 | 0.76 ± 0.15 | T-test | 0.92 |
| ***OCT (µm)*** | 70.05 ± 18.10 | 66.74 ± 19.71 | T-test | 0.53 |
| ***Phakic/pseudofakic (%)*** | 13/6 (68/32) | 27/12 (69/31) | Chi-sq | 0.86 |
| ***Family history (Yes/No) (%)*** | 10/9 (52/48) | 15/24 (38/62) | Chi-sq | 0.25 |
| ***Diabetes (Yes/No) (%)*** | 2/17 (11/89) | 8/31 (20/80) | Chi-sq | 0.45 |
| ***Hypertension (Yes/No) (%)*** | 9/10 (47/53) | 20/19 (51/49) | Chi-sq | 0.57 |
| ***Smoking (Yes/No) (%)*** | 6/13 (32/68) | 16/23 (41/49) | Chi-sq | 0.21 |
| ***MD at diagnosis (dB)*** | -3.87 ± 3.54 | -6.11 ± 4.58 | T-test | 0.04* |
| ***VFI at diagnosis (%)*** | 92.21 ± 9.88 | 88 ± 11.01 | T-test | 0.02* |

IOP: Intraocular Pressure. CCT: Central Corneal Thickness. C/D ratio: Cup/Disc ratio. OCT: Optical Coherence Tomograph. MD: Mean Deviation. VFI: Visual Field Index. (*) Significant values p=<0.05
