# Supplementary material for: Risk factors for visual field progression during 10-year follow-up in newly diagnosed exfoliation glaucoma patients
Source: Sci Rep. 2026 Jun 30;16:19909. doi: 10.1038/s41598-026-60254-x (PMC13319220; doi:10.1038/s41598-026-60254-x)
Supplement: Supplementary file 2 — Supplementary Material 2 [file 41598_2026_60254_MOESM2_ESM.docx]

Table 2-Supp

Distribution of MD values and slopes according to years of follow-up.

| **Year** | **MD absolute values**  **(dB) (SD)** | **MD slope**  **(dB) (SD)** | **MD slope < 1 dB**  **(N) (%)** | **MD slope ≥ 1 dB**  **(N) (%)** |
| --- | --- | --- | --- | --- |
| 0 | -5.38 (4.37) |  |  |  |
| 1 | -6.05 (4.52) | -0.67 (1.43) | 42 (72) | 16 (28) |
| 2 | -7.13 (4.88) | -1.08 (1.68) | 30 (52) | 28 (48) |
| 3 | -7.99 (4.94) | -0.86 (1.76) | 36 (62) | 22 (38) |
| 4 | -8.69 (5.37) | -0.7 (1.97) | 42 (72) | 16 (28) |
| 5 | -9.41 (6.07) | -0.72 (2.12) | 45 (78) | 13 (22) |
| 6 | -9.78 (6.34 | -0.37 (1.97) | 42 (72) | 16 (28) |
| 7 | -11.19 (6.71) | -1.41 (2.68) | 36 (62) | 22 (38) |
| 8 | -11.57 (7.13) | -0.38 (1.87) | 44 (76) | 14 (24) |
| 9 | -12.59 (7.91) | -1.02 (2.24) | 39 (67) | 19 (33) |
| 10 | -13.07 (7.92) | -0.48 (1.97) | 43 (74) | 15 (26) |

MD: Mean Deviation.
