# Supplementary material for: Risk factors for visual field progression during 10-year follow-up in newly diagnosed exfoliation glaucoma patients
Source: Sci Rep. 2026 Jun 30;16:19909. doi: 10.1038/s41598-026-60254-x (PMC13319220; doi:10.1038/s41598-026-60254-x)
Supplement: Supplementary file 3 — Supplementary Material 3 [file 41598_2026_60254_MOESM3_ESM.docx]

Table 3-Supp

Univariate analysis for predictors, endpoint MD values across ten years-follow-up.

| Predictor | Coefficient (B) | Coefficient 95% CI | P-value |
| --- | --- | --- | --- |
| Age | 0.35 | [0.14-0.56] | 0.002* |
| Central cornea thickness (CCT) | -0.06 | [-0.09;-0.01] | 0.007* |
| IOP at diagnosis | 0.35 | [0.09;0.61] | 0.009* |
| Phakia/pseudophakia (0/1) | -3.31 | [-6.91;1.81] | 0.06* |
| Smoking | 4.95 | [1.73;8.16] | 0.003* |
|  |  |  |  |
| Cataract surgery during 10 years | 1.3 | [-2.03;4.64] | 0.44 |
| Cup/Disc ratio (CD) | -4.36 | [-14.81;6.08] | 0.41 |
| Gonioscopy (pigment) | 1.94 | [-1.16;5.04] | 0.21 |
| Gonioscopy (Shaeffer) | 1.35 | [-1.85;4.56] | 0.41 |
| Heredity | -2 | [-5.33;1.32] | 0.23 |
| Hypertension | 1.80 | [-1.54;5.11] | 0.28 |
| Mean deviation (MD) at diagnosis | 0.01 | [-0.38;0.39] | 0.95 |
| Migraine | -1.45 | [-7.39;4.49] | 0.62 |
| OCT diagnosis | 0.005 | [-0.07;0.08] | 0.90 |
| Sex | 0.42 | [-2.92;3.76] | 0.80 |
| Spheric equivalent (SE) | -0.63 | [-3.30;2.31] | 0.63 |
| Unilateral presentation | 1.80 | [-1.72;5.32] | 0.31 |
| Visual Acuity (VA) | 3.42 | [-4.36;11.21] | 0.38 |
| VFI at diagnosis | -0.02 | [-0.16;0.11] | 0.72 |

(*) Significant values at p= < 0.10.
