# Supplementary material for: Risk factors for visual field progression during 10-year follow-up in newly diagnosed exfoliation glaucoma patients
Source: Sci Rep. 2026 Jun 30;16:19909. doi: 10.1038/s41598-026-60254-x (PMC13319220; doi:10.1038/s41598-026-60254-x)
Supplement: Supplementary file 4 — Supplementary Material 4 [file 41598_2026_60254_MOESM4_ESM.docx]

Table 4. Supp

Univariate analysis for predictors, endpoint VFI values across ten years-follow-up.

| Predictor | Coefficient (B) | Coefficient 95% CI | P-value |
| --- | --- | --- | --- |
| Age | 1.34 | [0.60;2.08] | <0.001* |
| CCT | -0.23 | [-0.37;-0.09] | 0.002* |
| IOP at diagnosis | 1.41 | [0.51;2.32] | 0.003* |
| Smoking | 17.73 | [6.38;29.08] | 0.003* |
|  |  |  |  |
| Cataract surgery during 10 years | 2.89 | [-8.97;14.75] | 0.62 |
| Cup/Disc ratio (CD) | -24 | [-60.75;12.53] | 0.19 |
| Gonioscopy (pigment) | 6.33 | [-4.67;17.34] | 0.25 |
| Gonioscopy (Shaeffer) | 3.54 | [-7.84;14.94] | 0.53 |
| Heredity | -6.65 | [-18.46;5.14] | 0.26 |
| Hypertension | 5.17 | [-6.57;16.91] | 0.38 |
| Mean deviation (MD) at diagnosis | 0.81 | [-0.53;2.15] | 0.23 |
| Migraine | -2.21 | [-23.77;18.44] | 0.83 |
| OCT diagnosis | 0.03 | [-0.26;0.32] | 0.83 |
| Phakia/pseudophakia (0/1) | -9.17 | [-21.71;3.36] | 0.15 |
| Sex | 3.37 | [-8.44;15.19] | 0.57 |
| Spheric equivalent (SE) | 0.72 | [-8.79;10.24] | 0.87 |
| Unilateral presentation | 2.95 | [-9.62;15.52] | 0.64 |
| Visual Acuity (VA) | 11.65 | [-15.93;39.23] | 0.4 |
| VFI at diagnosis | -0.32 | [-0.81;0.16] | 0.19 |

(*) Significant values at p= < 0.10.
