# Supplementary material for: Risk factors for visual field progression during 10-year follow-up in newly diagnosed exfoliation glaucoma patients
Source: Sci Rep. 2026 Jun 30;16:19909. doi: 10.1038/s41598-026-60254-x (PMC13319220; doi:10.1038/s41598-026-60254-x)
Supplement: Supplementary file 5 — Supplementary Material 5 [file 41598_2026_60254_MOESM5_ESM.docx]

Table 5- Supp

Univariate logistic regression analysis for predictors, endpoint GPA values across ten years-follow-up.

| Predictor | Coefficient (exp β) | Coefficient (exp β)  95% CI | P-value |
| --- | --- | --- | --- |
| Age | 1.23 | [1.09;1.38] | <0.001* |
| CCT | 0.99 | [0.97;1.0] | 0.09* |
| IOP at diagnosis | 1.18 | [1.05;1.32] | 0.005* |
| Smoking | 9 | [1.82;44.27] | 0.007* |
|  |  |  |  |
| Cataract surgery during 10 years | 0.74 | [0.25;2.22] | 0.59 |
| Cup/Disc ratio (CD) | 1.63 | [0.06;47.14] | 0.77 |
| Gonioscopy (pigment) | 1.32 | [0.48;3.63] | 0.59 |
| Gonioscopy (Shaeffer) | 1.88 | [0.62;5.66] | 0.26 |
| Heredity | 0.65 | [0.21;1.94] | 0.44 |
| Hypertension | 1.57 | [0.05;3.22] | 0.41 |
| Mean deviation (MD) at diagnosis | 1.08 | [0.94;1.23] | 0.28 |
| Migraine | 2.23 | [0.23;21.46] | 0.48 |
| OCT diagnosis | 0.98 | [0.96;1.01] | 0.29 |
| Phakia/pseudophakia (0/1) | 1.07 | [0.33;3.49] | 0.89 |
| Sex | 0.81 | [0.27;2.39] | 0.7 |
| Standard equivalent (SE) | 0.73 | [0.31;1.68] | 0.46 |
| Unilateral presentation | 0.57 | [0.17;1.91] | 0.36 |
| Visual Acuity (VA) | 0.73 | [0.05;9.58] | 0.81 |
| VFI at diagnosis | 0.96 | [0.92;1.06] | 0.23 |

(*) Significant values at p=<0.10
